# Pathological Thoracic Vertebral Fractures Secondary to Achromobacter denitrificans: A Case Report

**DOI:** 10.7759/cureus.59141

**Published:** 2024-04-27

**Authors:** Gerardo Romero-Luna, Natalia M Barron-Cervantes, Jimena Gonzalez-Salido, Jimena Colado-Martínez, Gustavo Anaya-Delgadillo, Pedro Pablo De Juambelz-Cisneros

**Affiliations:** 1 Neurosurgery, Hospital Español de Mexico, Mexico, MEX; 2 Medicine, Universidad Panamericana, Mexico, MEX; 3 Medicine, Universidad La Salle, Mexico, MEX

**Keywords:** spine fracture, vertebral infection, osteomyelitis, pathological fractures, achromobacter denitrificans

## Abstract

Vertebral fractures remain a diagnostic challenge nowadays. The first and most common diagnosis needed to be ruled out is osteoporosis. Other diagnoses to rule out involve pathological fractures. Pathological fractures are a group of pathologies that result in a spine fracture as part of an underlying disease process that affects the spine. This group includes Paget’s disease, tumors, osteomyelitis, and vertebral compression fractures. Fractures secondary to vertebral osteomyelitis are presented as collapsed vertebral bodies secondary to bone destruction and the formation of lytic lesions. Clinical presentation includes severe back pain refractory to analgesic therapy, persistent unexplained fever, and leukocytosis without any other obvious focus of infection. In cases like the one presented here, early biopsy and culture should be performed on every patient that fits these criteria. However, as it presents unspecific symptoms most of the time, it is not suspected, and therefore it is associated with high morbidity and mortality.

## Introduction

Pathological fractures include a group of pathologies that result in vertebral fractures secondary to an alteration in the bone physiology. Within this group, there are benign and malignant diseases. It is of great importance to rule out any type of malignant tumor, especially in cases where osteolytic lesions occur. The majority of pathological fractures are associated with metastatic disease, most commonly lung and breast primary tumors, rather than primary bone tumors. However, when it is a primary tumor, the most common is bone sarcoma [1]. Pathological fractures secondary to osteomyelitis are a rare complication of osteomyelitis in adults, with community-acquired methicillin-resistant Staphylococcus aureus (CA-MRSA) being the most common etiology, followed by Pseudomonas and Enterobacteriaceae. The primary source of dissemination to the vertebra is from the skin [2]. Systemic dissemination through hematologic or lymphatic routes, although not common, is possible. Hematogenous osteomyelitis in immunocompetent adults is very rare with only a few case reports reported worldwide. The key in this diagnostic approach is ruling out every other possible cause for the pathological fracture, such as metastatic bone tumors, multiple myeloma, primary malignant bone tumor, or Paget’s disease. Additionally, when establishing that the cause of the fracture is indeed osteomyelitis, the next important step is determining the microorganism causing the infection [3].

In cases like the one presented in this text, one of the most important tools used through the diagnostic approach is the use of bone biopsy accompanied by cultures and special antibody tests. Additionally, the presence of acute suppurative inflammation in which the microorganisms are embedded helps reach out to a final diagnosis by presenting antigens that may be tested directly. Complete evaluation in these patients also includes an imaging study, a contrasted magnetic resonance imaging (MRI) being the gold standard, and a set of laboratory tests, such as white blood cell (WBC) count and inflammatory markers [3]. The following review presents the case of a 65-year-old male who presented with lumbar pain refractory to analgesic per oral (PO) therapy 9/10 that worsens at movement, limiting his daily life activities in a first-level private surgical center in Mexico City. This case is presented in order to further expand the knowledge about pathological fractures, as well as highlight the importance of ruling out osteomyelitis in cases like these. It is important to emphasize that no other case in literature has presented a relationship between Achromobacter denitrificans and pathological fractures.

## Case presentation

A 65-year-old Latin American male presents to the outpatient clinic due to lumbar pain of three-month duration, located in the bilateral lumbar region 8/10, with no radiation of the pain, which increases in the supine position and no longer shows improvement with over-the-counter PO analgesic and limits his daily life activities. Something important to emphasize is that the patient mentions that there was no possible mechanism of injury that may have caused the pain. Regarding his past medical history, he is allergic to nonsteroidal anti-inflammatory drugs (NSAIDs) and sulfamethoxazole, and his father passed away because of laryngeal cancer. He was diagnosed with systemic hypertension in 2021, currently in treatment with amlodipine 5 mg PO every 24 hours and losartan 50 mg PO every 12 hours. Additionally, he was diagnosed with benign prostatic hyperplasia (BPH) in 2019 and is currently in treatment with dutasteride-tamsulosin 0.5/0.4 mg PO every 24 hours. Moreover, in 2019, the patient presented a traumatic fall in which he was diagnosed with Rockwood type V acromioclavicular right dislocation for which a right acromioplasty was performed. During the physical examination (PE), he presented with localized pain at the level of L1, no neurological alterations were presented, and he preserved his strength and sensation in all extremities. Pain limited deambulation. No other alterations were noted. By his own decision, the patient underwent a T2-weighted MRI of the lumbar spine where a non-displaced fracture at the level of L1 was reported. It is important to mention that he presented to the clinic with this report but did not present images. Due to his age and the fact that he did not present an injury mechanism, a pathological fracture was suspected, so it was decided to admit him for a study and treatment protocol.

**Figure 1:**
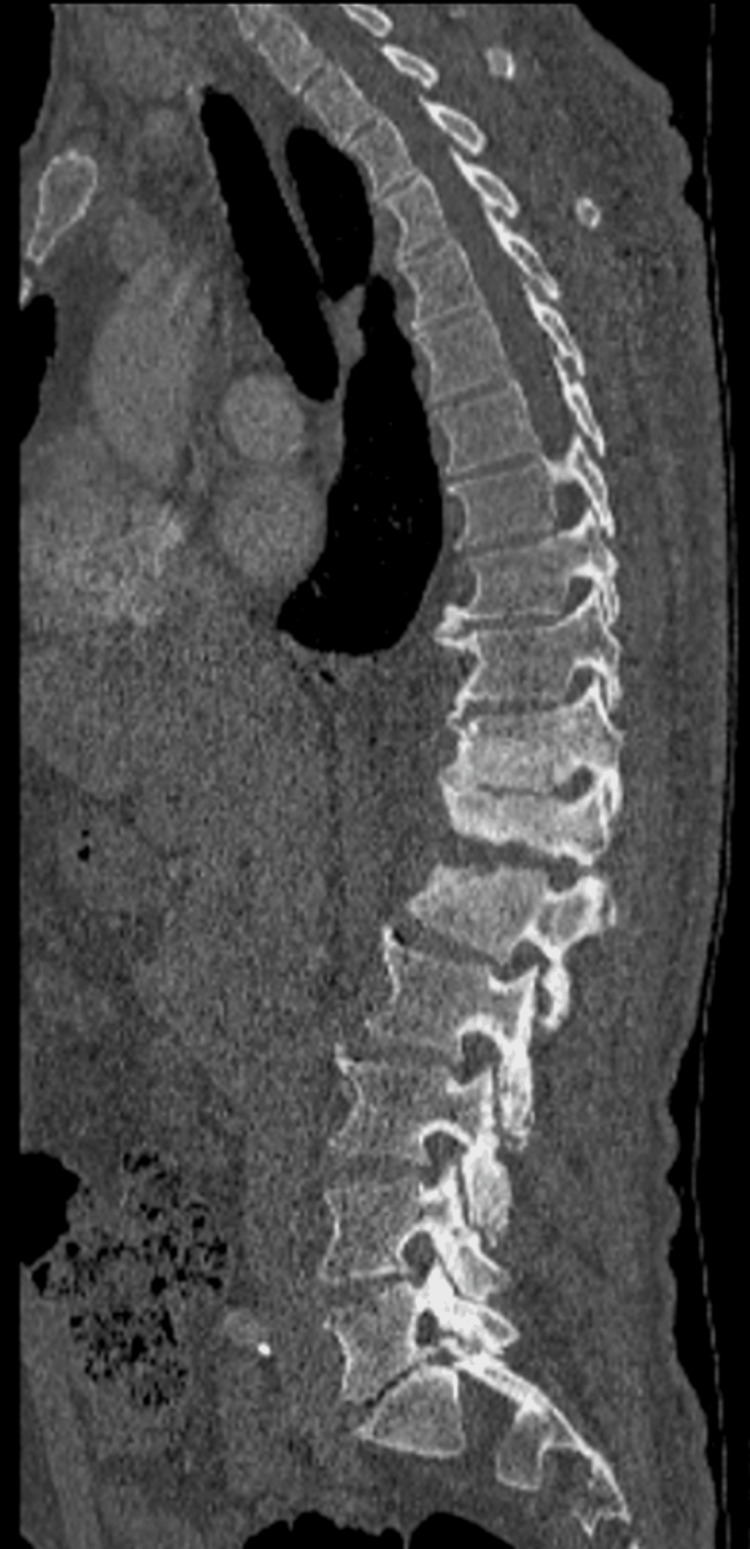
Non-contrasted lumbar spine CT scan. Lytic lesions at the level of T10, T11, T12, and L1. Loss of morphology in L1, T11, and T12. Loss of height T11-12. Adjacent collection in the psoas muscle on the left side and a mass that generates sclerosis in T11, T12, and L1.

During his stay, a non-contrasted lumbar spine CT scan of the spine was performed with the finding of lytic lesions at the level of T10, T11, T12, and L1. Loss of morphology in L1, T11, and T12 was also noted with loss of height T11-12, adjacent collection in the psoas muscle on the left side, and a mass that generates sclerosis in T11-T12 and L1 (Figure 1).

**Figure 2:**
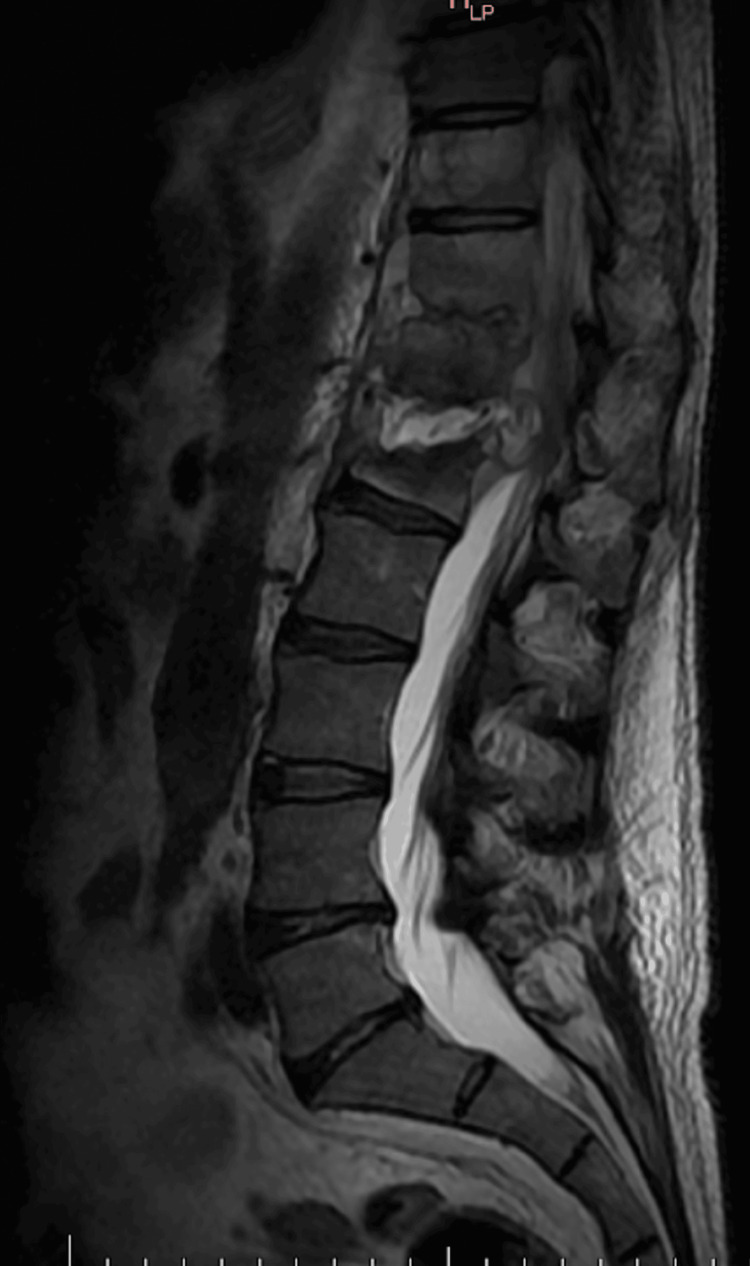
MRI T2-weighted sequence. High-intensity signal inside the L1-L2 disk on T2-weighted sequences and destroyed L1 vertebral end plate, accompanied with high-signal-intensity marrow edema. Additionally, a left psoas muscle abscess was noted.

**Figure 3:**
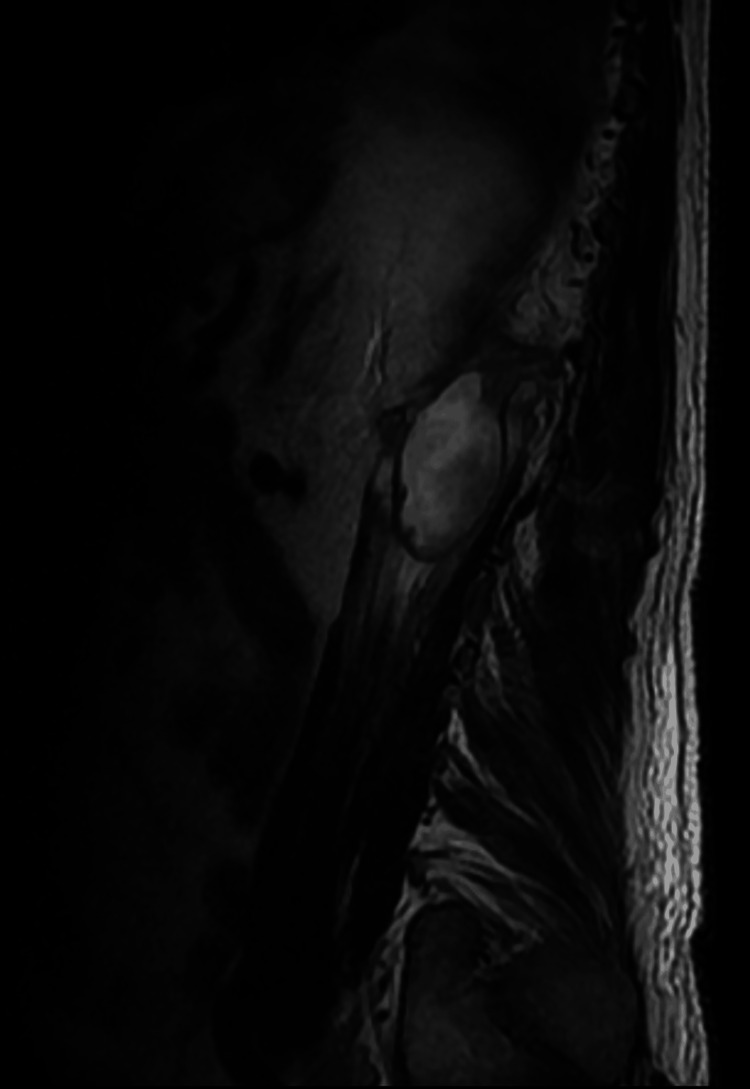
FLAIR MRI. Collection at the level of the psoas from T12 to L1 with well-defined borders and homogeneous content, confirming the presence of an abscess FLAIR: fluid-attenuated inversion recovery

During his first week of hospitalization, pain management improved owing to the administration of opioids, specifically tramadol 100 mg intravenously (IV) every 24 hours and acetaminophen 1 g IV every eight hours. However, the patient began presenting hemoptysis, vertigo, and nausea. When questioning the patient, he mentioned that he had had episodes of nocturnal cough for the last three months but never had hemoptysis. Based on the clinical situation, the decision was made to perform a mycobacteria culture in expectoration fluid, as well as a polymerase chain reaction (PCR) for tuberculosis in the said sample. Additionally, due to the findings of the CT scan and bone scan, the decision was made to perform a bone biopsy at L1 using general anesthesia in the operating room (OR). Culture for fungi, bacteria, mycobacteria, auramine rhodamine, Grocott stain, Ziehl Neelsen stain, and PCR for tuberculosis were performed on the sample obtained from the biopsy. In addition, a gallium uptake scan was performed in the lumbar spine where it was reported to be suggestive of infection, presenting with a high-intensity signal inside the L1-L2 disk on T2-weighted sequences and destroyed L1 vertebral end plate, accompanied by a high-signal-intensity marrow edema. Additionally, a left psoas muscle abscess was noted (Figure 2), Therefore, a fluid-attenuated inversion recovery (FLAIR) MRI was also performed where the presence of a collection was observed at the level of the psoas from T12 to L1 with well-defined borders and homogeneous content, confirming the presence of an abscess (Figure 3). Similarly, due to poor pain management metamizole 1 Gram IV every 24 hours as an infusion was added to the solution for pain control.

The patient presented with exacerbating low back pain and respiratory symptoms, prompting a request for antibodies against Brucella. Subsequently, empirical antibiotic therapy was initiated, comprising ertapenem administered intravenously at a dosage of 1 g every 24 hours, along with vancomycin administered intravenously at a dosage of 1 g every 12 hours. Additionally, analgesic treatment was updated with buprenorphine transdermal patch 10 mg subcutaneous (SC). The pulmonology team added nebulizations with ipratropium bromide every eight hours and budesonide every 12 hours. Similarly, due to the persistence of symptoms, it was decided to perform a thoracic vertebra biopsy under general anesthesia in the OR. All the previous cultures were repeated for this new biopsy, just adding a new culture for Achromobacter denitrificans, which came out positive.

Based on the microbiology results, it was decided to change the antibiotic management to ceftazidime 2 g IV every eight hours, rifampicin 300 mg IV every 12 hours, and trimethoprim/sulfamethoxazole 160/800 mg IV every eight hours. Since antibiotic management began, the patient began to decrease presenting respiratory symptoms until he was discharged by the pulmonology team. However, during his first week with this new treatment, he developed a generalized maculopapular rash that blanched with acupressure that was associated with the administration of trimethoprim/sulfamethoxazole, which was why it was changed with meropenem 1 g IV every eight hours. Finally, the decision was made to drain the left psoas muscle abscess by lumbotomy. Eventually, the patient continued with analgesic management based on transdermal patches of fentanyl 25 mcg/hour and pregabalin 75 mg PO every 12 hours. The antibiotic management scheme is completed, and it is decided to discharge the patient for external follow-up.

## Discussion

Pathological fractures are a bone pathology in which a bone fracture is produced as a result of an altered skeletal physiology associated with a benign or malignant lesion. The primary concern that warrants consideration is the potential presence of musculoskeletal malignant tumors and metastatic disease, owing to the significant morbidity and mortality they entail [1]. The most common cause of pathologic fractures is malignant tumors, metastatic disease being more frequently presented than primary bone tumors, such as sarcoma [4]. The primary cancers that must be ruled out when presenting metastatic diseases are lung, breast, thyroid, renal, and prostate cancer with the most common anatomical regions affected being the spine, proximal femur, and pelvis [5]. Pathophysiologically, osteolytic lesions secondary to malignant tumors are associated with a tumor-induced upregulation of RANK ligand (RANK-l) by endothelin 1, which constitutionally activates osteoclasts, thus generating small lytic lesions that when placed under low-demand activity can cause a complete breakdown of the bone, resulting in what is known as a pathological fracture [6]. A bone biopsy should be performed in order to establish the final etiological diagnosis, as the histology seen will indicate whether it is a primary bone malignancy or metastasis, also in cases where malignant pathology is not the cause of the pathological fracture [7]. A bone biopsy will confirm that there is no malignancy and that bone samples can be sent for further studies, such as bone cultures in order to rule out other benign pathologies, such as osteomyelitis.

Vertebral osteomyelitis is a rare infectious pathology with a prevalence estimated at 2.4 cases per 100,000 population in the United States, associated with a higher rate of presentation among elderly people [8]. The most common causes presented are hematogenous seeding, direct infection during open spinal surgery, or contiguous spread from an infection in the soft tissue surrounding the spine. The most common microorganism implicated is Staphylococcus aureus associated with bacteremia. Other commonly seen pathogens are Staphylococcus epidermidis, Pseudomonas aeruginosa, Enterobacter spp., Nocardia spp., Actinomyces spp., and atypical Mycobacterium [8]. In cases where hematogenous seeding is suspected or proved by the diagnosis of bacteremia, the primary focus more frequently presented includes the urinary tract, endocarditis, bursitis, and septic arthritis [9]. In the literature, the most common initial symptom associated with vertebral osteomyelitis is vertebral pain, accompanied by fever and peripheral neurological impairment, such as weakness, paresthesias, or radiculopathy [8]. Diagnosis is suspected in patients who present spinal pain that does not improve with over-the-counter PO analgesics associated with leukocytosis with a high percentage of neutrophils, which is more than 80%. However, these do not present enough sensitivity to the diagnosis of osteomyelitis. Other associated laboratory findings can be the elevation of the erythrocyte sedimentation rate (ESR) and C-reactive protein (CRP), and these present a sensitivity from 98% to 100%. However, these present a low specificity for this diagnosis [10].

Blood cultures are crucial in the evaluation of these cases because of their having a hematogenous origin. Diagnosis of vertebral osteomyelitis is based on the positive bone culture. However, the most commonly non-invasive image study used is the vertebral MRI, with an accuracy of over 90%. The typical image presents a high-intensity signal inside the disk on T2-weighted sequences, and vertebral end plates can be seen destroyed, accompanied by high-signal-intensity marrow edema [8]. For the bone culture and biopsy of the specimen, a CT-guided biopsy is the preferred method. Nevertheless, an open biopsy technique can be performed in the OR. It is important to mention that, if the CT-guided biopsy is negative, an open biopsy should be performed before finishing the infectious diagnostic approach [11]. All bone samples should be cultured for aerobic bacteria, anaerobic bacteria, fungi, mycobacteria, and brucella species. Additionally, the same bone sample should be studied by the pathology team in order to determine the presence of leukocytes or data compatible with acute inflammation in order to distinguish whether it is an active bone infection or contamination [11]. It is imperative to note that, if antibiotic therapy has commenced and the patient's condition remains clinically stable, deferring a biopsy for a minimum of 48 hours after the last administration of antibiotics can enhance its diagnostic yield. Although an antibiotic-free interval of one to two weeks could potentially yield better results, for safety considerations, it is generally not recommended in cases of acute osteomyelitis [8].

Antibiotic treatment, when possible, should always be based on sensibility tests against the identified microorganism. In cases where an acute hematogenous origin can be proved, antibiotic therapy by itself will be treatment enough. Surgery is required mostly for open biopsies and abscess drainage. The only cases where surgical treatment is indicated are when surgical implants presented are infected and must be removed and in cases where there is necrotic tissue that must be debrided [12]. Achromobacter denitrificans is an uncommon opportunistic bacteria, which is a strictly aerobic, motile, and non-fermenting Gram-negative bacilli that forms part of the Alcaligenaceae and order Burkholderiales. This is a rare saprophytic bacteria that is mostly seen in healthcare-associated infections [13]. In worldwide literature, only one review by Imani et al. [14] reported six cases associated with Achromobacter as the cause of osteomyelitis. For this species, empiric therapy with carbapenems is recommended. However, as acquired resistance is increasingly being reported, antibiotic susceptibility testing should be performed in all cases when possible as it is essential for therapy. In addition, surgical drainage and debridement should be performed when indicated, as previously mentioned [13].

## Conclusions

This case illustrates one of the rarest presentations encountered in the field of medicine: osteomyelitis associated with Achromobacter denitrificans. It underscores the challenges inherent in its diagnosis, given its uncommon pathogenicity, thereby necessitating delayed diagnosis and tailored antibiotic management guided by susceptibility testing. The case highlights the nonspecific clinical manifestations compounded by multiple negative antimicrobial tests, further complicating the accurate diagnosis. With only limited instances documented in the global medical literature, this case offers valuable insights for learning and preventive measures for future patients presenting with vertebral osteomyelitis devoid of commonly associated pathogens. In conclusion, when faced with diagnostic ambiguity, it is paramount to consider pathogens uncommonly linked with the condition or those seldom encountered in clinical practice.
